# Australia II: A Case Study in Engineering Ethics

**DOI:** 10.1007/s11948-024-00477-1

**Published:** 2024-05-08

**Authors:** Peter van Oossanen, Martin Peterson

**Affiliations:** 1 Van Oossanen Naval Architects, Nude 46, 6702 Wageningen, DM The Netherlands; 2 Department of Philosophy, Texas A&M University, College Station, TX 77843 USA

**Keywords:** Wing keel, America’s Cup, NSPE Code of Ethics, KIVI Code of Ethics

## Abstract

Australia II became the first foreign yacht to win the America's Cup in 1983. The boat had a revolutionary wing keel and a better underwater hull form. In official documents, Ben Lexcen is credited with the design. He is also listed as the sole inventor of the wing keel in a patent application submitted on February 5, 1982. However, as reported in *New York Times*, *Sydney Morning Herald*, and *Professional Boatbuilder*, the wing keel was in fact designed by engineer Peter van Oossanen at the Netherlands Ship Model Basin in Wageningen, assisted by Dr. Joop Slooff at the National Aerospace Laboratory in Amsterdam. Based on telexes, letters, drawings, and other documents preserved in his personal archive, this paper presents van Oossanen’s account of how the revolutionary wing keel was designed. This is followed by an ethical analysis by Martin Peterson, in which he applies the American NSPE and Dutch KIVI codes of ethics to the information provided by van Oossanen. The NSPE and KIVI codes give conflicting advice about the case, and it is not obvious which document is most relevant. This impasse is resolved by applying a method of applied ethics in which similarity-based reasoning is extended to cases that are not fully similar. The key idea, presented in Peterson’s book *The Ethics of Technology* (Peterson, The ethics of technology: A geometric analysis of five moral principles, Oxford University Press, 2017), is to use moral paradigm cases as reference points for constructing a “moral map”.

## Introduction

The America’s Cup is the world’s most prestigious sailing competition. It has been sailed regularly since 1851, which makes it the oldest continuous international sporting competition. In 1983, *Australia II* became the first foreign yacht to win the Cup after beating the American defender, *Liberty*. The Australian victory ended what is still the longest winning streak in sports history—132 years! After the final race, Australian Prime Minister Bob Hawke got so excited that he effectively encouraged all citizens to take a day off from work to celebrate: “Any boss who sacks a worker for not turning up today is a bum!".^1^

**Fig. 1:**
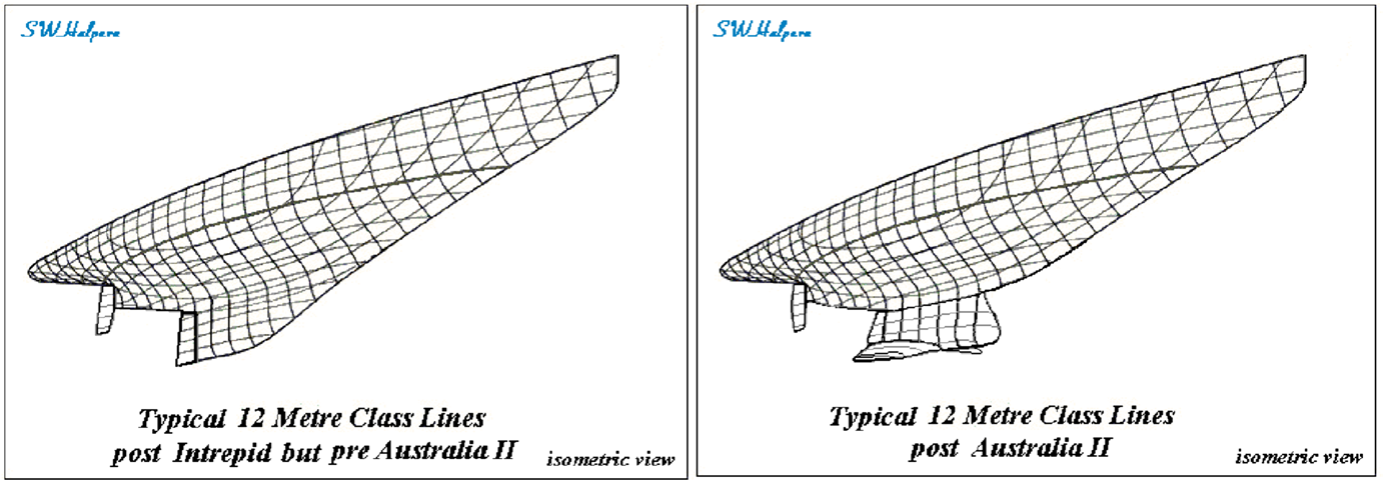
The drawing to the left depicts a traditional keel. *Australia II’s* keel is to the right. (Reprinted with permission from Samuel Halpern)

*Australia II* did not win because the Australian team sailed better. Dennis Connor, the American skipper of *Liberty*, is widely recognized as the best sailor of all time. *Australia II* won because of a revolutionary wing keel and a better underwater hull form, which gave it a considerable edge over the more conventional boat designed by the American defenders. See Fig. 1.

The wing keel was novel in two respects: (i) The bottom of the keel was longer than the top. This “inverted” keel resulted in a lower center of gravity, which led to a stiffer boat that sailed faster and more upright against the wind. (ii) The keel was equipped with winglets designed to reduce drag (the resistance of the boat moving through water). Just like winglets on modern airplanes reduce wingtip vortices, winglets on the keel reduce the swirling of water at the trailing edge of the lower tip.

At the time, the rules of the America’s Cup mandated that boats must be designed by nationals of the country they represented, meaning that an Australian boat had to be designed by an Australian designer. In official documents, as well as in the Netflix documentary”Untold: The Race of the Century” (2022) and in *Encyclopedia Britannica*, the Australian designer Ben Lexcen (1936–1988) is credited with the design of *Australia II* and its revolutionary wing keel.^2^ Lexcen is also listed as the sole inventor in a patent application submitted on February 5, 1982.^3^ However, none of this is true. Lexcen left school at the age of fourteen, he had no formal education, and did not know how to operate a computer.^4^ As reported in several newspaper articles in *New York Times*, *Sydney Morning Herald*, and *Professional Boatbuilder*, the wing keel was in fact designed by the Dutch engineer Dr. Peter van Oossanen (b. 1943) at the Netherlands Ship Model Basin in Wageningen, assisted by Dr. Joop Slooff at the National Aerospace Laboratory in Amsterdam.^5^

Slooff (2016) has presented his account of how the keel was designed in a self-published book. However, the key person was van Oossanen. Based on telexes, letters, drawings, and other documents preserved in his personal archive, this paper tells van Oossanen’s side of the story in his own words.^6^ The scholars asked to review the text for *Science and Engineering Ethics* have been given access to an extended version of van Oossanen’s account (about 25,000 words) as well as copies of letters, drawings, and telexes. van Oossanen’s presentation is followed by an ethical analysis by Martin Peterson, in which he applies the American NSPE and Dutch KIVI Codes of ethics to the information provided by van Oossanen. Both authors are jointly responsible for this introduction; van Oossanen is solely responsible for the presentation of facts in Sect. "Peter van Oossanen’s Account of the Design Process", and Dr. Peterson is solely responsible for the ethical analysis in Sect. "Martin Peterson’s Ethical Analysis".

Both the NSPE and KIVI codes were violated on several occasions. However, the NSPE and KIVI codes evaluate some aspects of the case differently. To overcome this ethical impasse, we apply a method of applied ethics based on Aristotle’s observation that one should “treat like cases alike” in which similarity-based reasoning is extended to cases that are not fully similar. The key idea, presented in Peterson’s book *The Ethics of Technology* (2017), is to use moral paradigm cases as reference points for constructing a “moral map”.

## Peter van Oossanen’s Account of the Design Process

I met Ben Lexcen and Alan Bond for the first time at the Sebel Townhouse Hotel in Sydney, in the afternoon of January 19, 1978. I had written a letter to Alan two weeks earlier, introducing myself and explaining that I had just completed a study on how to optimize the design of Twelve Meter Class yachts for Newport, Rhode Island, wind conditions. In the letter, I hinted at the possibility of being able to improve the performance of *Australia*, Alan Bond's Twelve Meter which had competed in the America's Cup four months earlier. I also pointed out that I was a believer in using large-scale models for determining performance differences between different designs accurately. I explained that I worked at the Netherlands Ship Model Basin, that I had grown up in Sydney, and that I was keenly interested in yacht design.

The first thing Alan wanted to know was the result of my analysis of *Australia's* loss against *Courageous* in the 1977 America's Cup, which still bothered him. I explained that the keel needed improving because *Australia* lost ground against *Courageous* upwind. Downwind, *Australia's* performance was fine. Alan agreed. He became very amicable at that point, and I concluded that he was in agreement with what I told him. I then explained more fully who I was and what tank testing at NSMB was to entail. I explained that the secret of model testing when small speed differences between designs are important is the use of large models, and I proposed that we adopt one-third scale models. Such models had never been used before. Alan then asked me about the costs of model testing and I showed him an example test program that I had prepared in anticipation. He also asked me if I was prepared to work with Ben Lexcen on the project. I explained that I would be happy to work with Ben or anyone else that he wanted me to work with. We then discussed in detail what we could bring to his effort—both in terms of experimental testing and design.

On February 22, one month later, I still hadn’t heard from either Ben or Alan. I wrote a letter to Alan, asking him about his plans for the 1980 America’s Cup. When, a few weeks later, there was still no answer, and when my telephone messages were to no avail, I decided to approach other syndicates.

I need to make mention here of the fact that the managing director of NSMB at the time, Dick van Manen, supported me in every way in attracting an America’s Cup team to carry out research at NSMB. He, and Rinus Oosterveld, Head of Research and Development and my immediate superior, knew that to be successful, significant resources needed to be set aside to update the NSMB test set-up for towing models at varying angles of heel and leeway, for the analyses of the resulting test data, and for the extrapolation of that data to full-scale to derive performance predictions for the actual yacht.

Three years later, out of the blue, Ben Lexcen called me on Saturday, January 31, 1981. He was in Amsterdam and proposed driving to Wageningen to see the NSMB facilities. I could tell that he was impressed with what he saw when we walked through each of the seven laboratories. He told me that Alan was now convinced that to continue his quest for the America’s Cup he needed to build a new Twelve. He asked me to prepare a formal proposal that he would forward to Warren Jones, managing director of Alan Bond’s Challenge organization. Due to travel abroad, I wasn’t in a position to prepare the proposal until February 17. I sent it by regular mail, not believing that it would lead to an order, but when Ben sent me a telex about not having received anything, on February 20, I concluded that, this time, the acquisition of an America’s Cup project would not lead to a wild-goose chase.

### Testing Begins

A formal order for the program discussed in Sydney on March 13 arrived by letter dated March 17. We were given authority by Warren Jones to modify the program as required, allowing the cost to vary accordingly. He demanded “maximum security of all information compiled for us”.

As it happened, I had met Joop Slooff, an aerodynamicist employed by the National Aerospace Laboratory (NLR) in Amsterdam, more than a year ago. We were both seconded to a Royal Netherlands Navy working group studying the merits of employing advanced marine vehicles for the tasks the Navy stood for. We discovered we had similar interests, one of which was sailing. I called him on April 13, to ask him about the possibility of performing calculations to determine the merits, in terms of lift and drag, of widely differing keel shapes. He was enthusiastic and immediately agreed to perform the study. On informing me of the approximate cost, I sent a telex to Warren and Ben on April 15, as follows: “A new computer program has become available at the aerospace laboratory in Amsterdam with which a study can be carried out to determine the optimum shape of the keel for maximizing side force and minimizing the associated (induced) drag. I strongly recommend that this program be used in arriving at one of the alternatives to be tank-tested. Please forward your agreement, or otherwise, by return telex.”

The extensive series of tests with the base model, finished on May 6, 1981. The analyses of the results required two days. These were discussed with Ben on Friday morning, May 11. Ben and Yvonne checked out of their hotel afterward to tour the country. Ben returned to NSMB together with Warren Jones on Friday, May 15. The three of us spent time on the towing carriage that day and the next (on Saturday), discussing the project and the results obtained thus far, after which Warren left to return to Australia.

Joop Slooff had meanwhile completed the calculations for four “planar” keels, i.e. the keels without winglets. He called me on May 12, 1981, to say that the “inverted” keel I had proposed (with zero sweep-back and a taper ratio equal to two) was marginally better than Ben’s traditional keel, leading to approximately a 1% increase in velocity made good to windward in 8 knots of true wind. The results of Joop’s calculations were discussed at his office at NLR on Tuesday, May 19, with Ben attending. Joop expanded on what he told me over the phone on May 12. Ben and I agreed with Joop’s suggestion to add winglets to the panel model of the inverted keel and to repeat the calculations. We discussed the design of these winglets in terms of location, span, chord length, and dihedral angle.

Joop called me on June 5, 1981. He was over the moon when he told me that the winglets had reduced the drag due to lift (the induced drag) by as much as 35% in the computer simulation. I was relieved to have been told that because we had already prepared the drawing of the winglets, as an add-on to the inverted keel, and the manufacture thereof had commenced. Ben had left the country earlier, to spend a week in London. He there discussed the construction of the yacht with Lloyds Register.

The model fitted with the inverted keel was tested in the towing tank on Monday, June 9. The winglets were added late that evening and the tests with the resulting configuration were carried out the following day, June 10, 1981. Ben was present on both days, and I invited Joop to witness the tests with the winglets fitted. Our eyes were glued to the raw data the computer on the towing carriage spat out after each run. That information told us that the generated lift (side force), was significantly higher, but the drag was as well. The winglets had added wetted surface area, leading to higher friction drag. It became obvious to all of us that afternoon that the keel needed to be considerably smaller, retaining its depth but decreasing its chord length. My graph of measured side force versus leeway angle led me to propose a reduction of 25%. When that was decided my draftsman was given instructions on how the keel and winglets needed to be modified. Ben prepared a sketch of the way the keel would look. I released that sketch to journalists after the Cup was sailed.

The results in terms of the speed made good to windward, available the following day, showed disappointing results. When I showed the results to Ben, he too was disappointed—a let-down after seeing the high side force values the keel with winglets was developing the previous day.

The project by now had taken on a considerable increase in scope and required budget. Warren again agreed to cover the cost. In his telex to me dated June 9, he informed me as follows: “… I agree that Lexcen must remain [at NSMB] until final configuration agreed, thus leaving you to complete your formal report which is required by Alan Bond by the earliest possible date. We are becoming concerned that our total program is already six weeks behind our original schedule. …” In this telex Warren alluded to the importance of Ben’s attendance at NSMB for the duration of the project. I had informed him before that Ben was “sick of cheese” and that he wanted to return to Australia as soon as possible. It was the first time in all of our discussions and correspondence that Warren and I hinted at the Dutch influence on the design of the yacht and its possible consequences. This was later to become an issue, leading the NYYC to consider abandoning the 1983 America’s Cup. Ben didn’t heed Warren’s instruction and, after providing him with copies of our drawings, calculations, and test results, left NSMB on June 17, 1981.

**Fig. 2:**
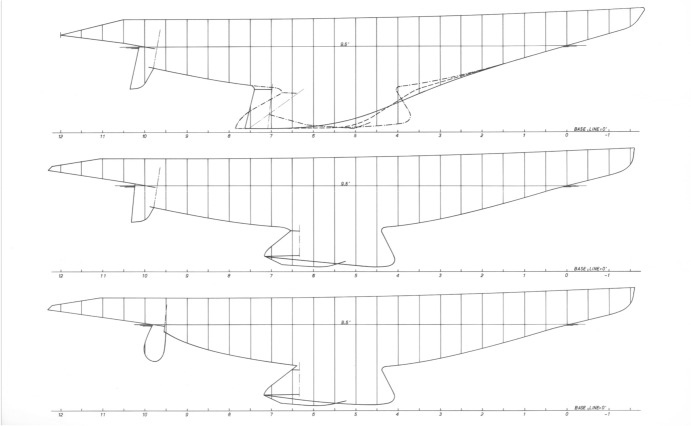
Hull, keel, trim tab, and rudder contours of the configurations that were tested. The continuous line in the top drawing is the contour of the original “Australia” model. The contour in the middle figure is that of the first refined inverted keel on the original “Australia” hull and that of the lower figure is that of the final configuration (the rudder was later modified)

Wednesday, July 1 through to Friday, July 10, was to become the high point of the project for me. Not having Ben or Joop around to care for, I sat down with my draftsman and redesigned the aft 40% of the hull. (See Fig. 2.) This entailed a considerable modification, consisting of removing the pronounced bustle that all previous Twelves possess since *Intrepid*, and moving the rudder stock forward by 0.3 m. I increased the thickness of the winglets (to fit more lead ballast) and reduced the height of the keel to that required by the Rule when waterline length is lessened. I took the liberty of changing the design of the rudder and trim tab as well. I sent Warren a long letter on July 2 explaining what we were doing. I called Ben on July 4 to update him on the test results and sent him a long telex on July 6 explaining that what we were doing was necessary to improve light-air performance. In the letter to Warren, I addressed the cost and time issues. Knowing that I needed to explain my reasoning in detail, I decided to travel to Australia to discuss everything and answer questions.

I travelled to Sydney on Tuesday, July 14, and visited Ben at home in Seaforth on Thursday, July 16. I showed him the results of the last set of tests, calling his attention to the results of upright drag tests (representing downwind conditions) which revealed the boat was slower than the base boat in light conditions. Both Warren and Ben agreed with my re-design proposals which included modifications to keel and winglets. As I was responsible for other projects in Australia, I travelled home on Friday, July 24, and returned to my office on Monday, July 27. I was surprised when I received a message from Warren Jones that morning about wanting to visit us together with Alan Bond and helmsman-elect John Bertrand, and that they would arrive within the hour. He explained that they were sailing the Admirals Cup (in England) and that today was a lay day. They had travelled to Wageningen by helicopter. They wanted to see the latest model and talk about its performance.

**Fig. 3:**
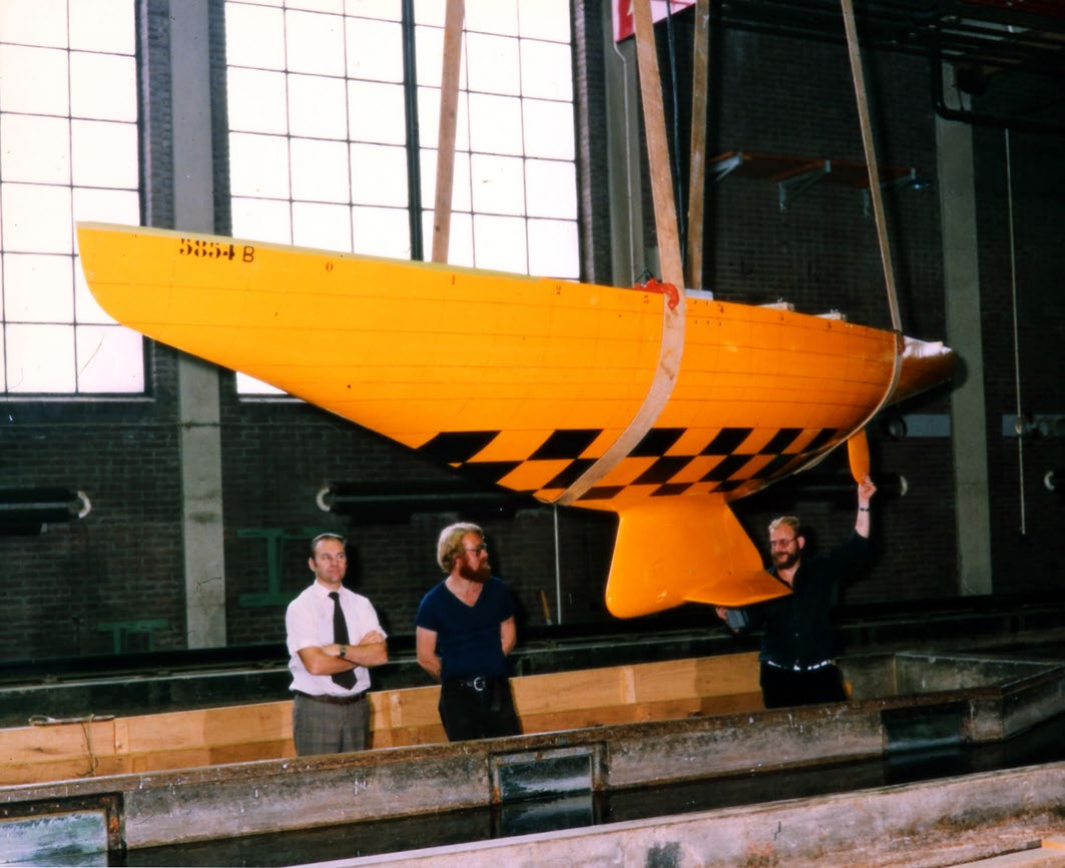
Photograph taken in the morning of August 6, prior to testing the final hull, keel, winglets, trim tab, and rudder. The two men on the right were the test technicians responsible for the tests that day and the next

The tests with the final hull fitted with the final keel and winglets, and the new trim tab and rudder, were performed on Thursday through Friday, August 6 and 7. See Fig. 3. I called Ben that Friday to inform him of the results. I told him we now had the performance needed to win the America’s Cup. See Table 1.

**Table 1:** Predictions of the speed made good to windward (in knots) based on tests with one-third scale models in the towing basin

True wind speed (knots)	Base *Australia* model	Base *Australia* model with inverted keel and winglets	Final hull with modified keel and modified winglets
5	3.26	3.26	3.34
6	3.92	3.88	3.98
7	4.46	4.45	4.52
8	4.90	4.97	5.04
9	5.34	5.45	5.52
10	5.70	5.81	5.88
11	5.99	6.09	6.15
12	6.22	6.31	6.37
13	6.41	6.49	6.55
14	6.56	6.65	6.69
15	6.69	6.78	6.82
16	6.80	6.89	6.91
17	6.89	6.97	7.00
18	6.96	7.05	7.07
19	7.02	7.11	7.13
20	7.07	7.15	7.18

### The Patent Application

I received a telex from Warren Jones on August 11, 1981, advising me that he had instructed their lawyers to investigate the possibility of registering a patent on the keel and that Michael Boud from Parker & Parker in Perth would be contacting me to discuss this. Instead, I received a letter dated September 1, 1981, from Michael that he would be visiting me in Wageningen. The meeting took place on September 22, 1981. His report on the meeting underscores the need to take protective measures to prevent the keel from being copied by others. I warned him during the meeting that the necessity of disclosure to the public, generally 18 months after filing or, in some countries, 18 months after the patent has been granted, could possibly cause a problem. That would be the case if, for example, the patent wasn’t granted in the USA and was disclosed to the public before the application could be retracted or otherwise be allowed to lapse. I therefore advised him not to apply for a patent because of the risks involved. Michael Boud, however, was heavily influenced by the patent attorney he talked to in The Hague after our meeting (Mr. F. Veldhuijsen from the Nederlandsch Octrooibureau), resulting in his decision to propose to Warren that he should have a patent drawn up and filed. Warren concurred. Michael’s report to Warren Jones, dated September 25, 1981, includes the following paragraph:

The Dutch connection with the invention is undeniable and Ben Lexcen would be first to admit that the invention was a team effort. Whilst Peter van Oossanen and NSMB are perfectly happy about Ben Lexcen being designated the inventor, the contributions of NSMB and NLR and the various back-up facilities at both these organizations have been crucial in developing the keel. However, the situation is analogous to an individual making a discovery whilst in the employment of a company which supports him, and both Dutch organizations are aware of course that the joint effort was on behalf of their client Mr. Bond and his organization. Whilst it is desirable to keep a low profile on the Dutch connection, it is perfectly logical however that the testing work should have been done at NSMB, which is one of the very few organizations outside the United States where such sophisticated testing and assessment is possible.

In his report, Michael Boud concluded that the patent needed to emanate from the Netherlands, but that the applicant be an Australian and that an Australian be shown to be the inventor. He proposed to lodge the patent application in the Netherlands first because if granted all other countries are bound to follow. He also proposed to take 2 months to complete the required paperwork (text, design description, claims, and drawings) so that if the patent is lodged on December 1, 1981, the need to publish will not arise until May 31, 1983, which, provided the invention can be kept secret until then, would be too late for a third party to capitalize on the idea. I took issue with him on this conclusion later, knowing that a similar keel could be built in the US well in time for America’s Cup racing in September 1983 on learning on June 1, 1983, what the keel looked like.

**Fig. 4:**
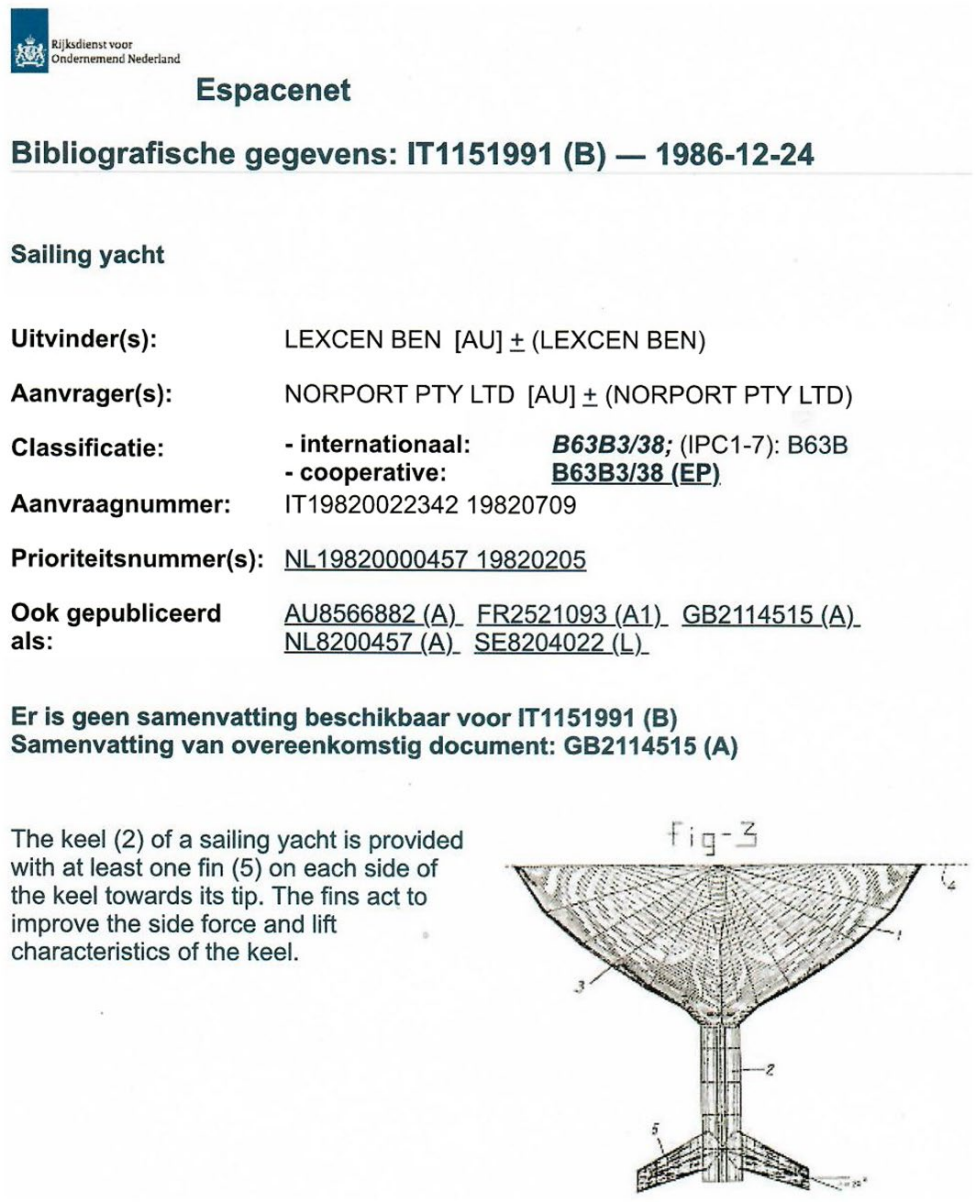
Excerpt from the Espacenet database of the patents files in the Netherlands (the priority filing) and Italy, Australia, France, Great Britain and Sweden

I received a draft of the patent application in Dutch together with a provisional translation to English, on December 3, 1981, as did Ben. Both Ben and I offered a host of comments and improvements. On January 25, 1982, Ben and I met Mr. Veldhuijsen in The Hague. We discussed the comments and improvements we had identified. My remarks about the Dutch application (the application to be filed) were also discussed. Ben had travelled by train from Amsterdam-Schiphol airport to The Hague. I drove him to the airport afterward, where we spent time discussing various projects he was busy with. The Netherlands application was filed on February 5, 1982, and disclosed on September 1, 1983. The applicant was Norport Pty. Ltd., a company incorporated in Australia. Norport was the company that acted as the trustee for a Discretionary Trust established by Ben for the benefit of his family. Ben was listed as the Inventor. See Fig. 4.

I received a long telex from Michael Boud on May 4, 1982. He informed me of “taking steps to instruct an attorney in Washington, DC, so that he will be in a position to head off any trouble which might arise in the US.” The patent attorney Michael referred to was Douglas (Doug) Henderson from Finnegan, Henderson, Farabow, Garret & Dunner, a reputable firm. I got to know Doug and his partner Steve Peterson. They assisted me later on various occasions with patent applications I filed with the United States Patent and Trademark Office (USPTO).

Filing of the patent application in the US was held up for months because Warren wanted assurance that the application wouldn’t be leaked by someone working for the US Patent and Trademark Office. In the end, Doug convinced Michael Boud that there was no risk of that nature. Meanwhile, it had been decided to place a copyright notice on all drawings and everything else pertaining to *Australia II*, including the yacht itself. We were asked to place that notice on all of our drawings and models. I informed Michael that everything concerning the design of the yacht was securely locked away and that such measures weren’t necessary at NSMB.

Doug and I decided to meet at his office in Washington, DC, on November 16, 1982. It fitted my itinerary perfectly because of scheduled meetings with personnel at the US Navy Laboratory in Carderock from November 15–17 (after I had left Perth on November 9, I flew to Sydney for meetings, then to Los Angeles, and from there to Washington, DC). Doug had been called away for another meeting when I arrived, and I spoke extensively with Steve Peterson and one of his colleagues. Knowing that nothing needed to be withheld, I answered all of his questions and provided all the data he needed.

In a follow-up telex dated November 22, Steve requested hard copies of various documents, and photographs of the last model we tested, to provide more accurate descriptions and drawings for the US patent. I was surprised to read: “… we will defer this study until we have further resolved any questions as to inventorship.” To preempt unwelcome discussions about inventorship, I sent Steve a telex on December 2 (and a copy to Michael Boud) as follows:

With reference to recent telexes and discussions concerning inventorship, I strongly recommend that a solution be sought in the fact that while research was being carried out, Lexcen, Slooff and I formed a team, of which Ben Lexcen was unquestionably the leader. As such, no discussion should arise as to which individual did what. The design was a team effort and Lexcen (as leader of the team) should be the applicant of the patent.

Doug sent me a long letter dated January 13, 1983, explaining the quandary we had been caught up in, as follows:

Thank you for your telex of December 2, 1982. The US patent laws require that an application for patent shall be made by the inventor. This language has been strictly construed by our courts to require that the inventor or inventors listed on the application must be the person or persons who actually made the invention which is set forth in the claims of the application. The courts have held that the head of a research team or department is not a proper inventor unless he has actually made the claimed invention himself. Where others working under the team leader’s direct supervision actually made the invention at his direction, the team leader will be considered the sole inventor if he suggested all the inventive features and the other members of the team were merely carrying out his specific directions. …. On the other hand, where the leader of the team only gives general instructions, and the team members actually conceived the specific features which are claimed in the patent application, then the team leader is at best a co-inventor with the member or members of the team who made the specific feature. … Finally, if the leader of the team merely instructs the team members to do studies to find ways to improve a device, and a team member comes up with a specific feature which makes the device better, then the team member is the sole inventor.

The patent issue was the lead into something more important. Warren explained that the patent issue had revealed that we (Joop Slooff, myself and my staff) were apparently not prepared to assume the role that he wanted us to take—that of never revealing what we did in the design of *Australia II*. He said that he had made it clear at the very start of the project when he visited NSMB that he wanted us to treat the project with the highest secrecy, which, to him, meant that we could never talk to anyone about what we were doing or what we were responsible for. I was surprised when he told me that because I had promised him exactly that, saying that NSMB frequently carried out projects of a confidential or secret nature about which we never talked about to anyone. My surprise was due to realizing that he doubted what I had then told him on behalf of not only myself and my staff but also on behalf of Joop Slooff, a subcontractor, whom I had told that the work he was to do was of a confidential nature. I there, in the presence of Warren and Ben, repeated my promise to never divulge the nature of what we had achieved. I added, however, that if *Australia II* were to win the America’s Cup it would hurt less if he and Ben were to be mindful of our role when explaining to the media how they had won—thereby implying that I would favor exercising discretion when speaking with the media. Both Warren and Ben agreed. We shook hands. I was not to know that Ben would pass on, on May 1, 1988, and Warren on May 17, 2002, and that, afterward, it appeared that no one on the *Australia II* team knew of what we were responsible for.

### The Summer of 1983

I arrived in Newport on June 17, 1983, to take charge of monitoring the yacht’s performance. The first sign of trouble surfaced on June 30, 1983, when I was confronted by Ben about having heard that Johan Valentijn (the designer of the American boat) knew all about the keel. In the evening of July 14, while talking to some of the crew, they showed me an article in a local newspaper. It covered an interview with Ben in which he alluded to having designed a revolutionary keel. The article quoted Ben as having said that the keel had wings. I showed the article to Warren who became quite upset. He confronted Ben in my presence about it. Ben shrugged his shoulders and said that everyone in Newport had already heard the keel had wings. He added that the information had originally come from Johan Valentijn.

On Saturday, July 16, Grant Simmer (navigator) showed me two articles, one in “Cruising World”, and one in a local newspaper. Both included sketches of what the keel might look like. One of these referred to Joop Slooff as having provided information about the keel to Johan Valentijn. I found it hard to believe but the proof was staring me in the face.

Joop Slooff called me in the afternoon on Wednesday, August 10. He had been visited by Antoon J. van Rijn and William Valentijn (a relative of Johan Valentijn) the day before. They wanted him (Joop Slooff) to admit that he had designed the keel of *Australia II*. When he wouldn't, they insisted on speaking to his superior. Since NLR had been engaged in the project as a subcontractor, he (Slooff’s superior) said he would refer the matter to NSMB. I was surprised when NSMB’s financial officer walked into my office not long after (I was back at NSMB for a few days during a short holiday with my family). He told me that he had been called by one of NLR’s directors (why would he call NSMB’s financial officer, I asked myself at the time). He wanted to know the reason Slooff and I wouldn’t acknowledge that we had designed the keel. I told him that if we did, the Australia II challenge would be banned from the America’s Cup competition.

Later that afternoon I received a call from Mr. K. F. Westerouen van Meeteren, a top official employed by the Ministry of Public Works, Transport, and Water Management. He had been visited by Van Rijn and Valentijn that morning. He wanted to be informed about the reason for not acknowledging that we had designed the yacht that looked like winning the America’s Cup. I explained the situation to him. When he asked me why we had accepted to carry out the work for the Australians in the first place, knowing that only an Australian national was allowed to design the yacht, I told him that NSMB’s management believed that it is not up to us to question the ethics of our principal. We simply carry out what we are asked to do. He then requested me to at least talk to the visitors from the US. I agreed. They called me a few minutes later, and I agreed to see them in my office at 6 p.m.

When Van Rijn and Valentijn arrived, I discovered that they were aware of what we had accomplished in 1981 in considerable detail. They had learned, they said, that Slooff had designed the keel. I decided not to make them any wiser and said that we had carried out what we were asked to do, describing the tests we did, without divulging who designed what. I explained that most projects carried out at NSMB are of a confidential nature, if not of a secret nature, such as the tests we do for the US Navy, which we do not talk about to anyone. Van Rijn and Valentijn left soon afterward.

I received a call from Warren that afternoon. He told me that he had been called by Joop Slooff several times the week before, and that Joop had threatened to formally protest if his name hadn’t been added as an inventor to the US patent. He asked me to talk to him “to calm him down”. Warren told me that he would withdraw the US patent if the matter couldn’t be contained. The patent was indeed withdrawn not much later.

I was visited by Richard S. Latham and William Valentijn on Wednesday, August 24, at two in the afternoon. They had scheduled an appointment earlier, and I had agreed to meet with them in accordance with Warren’s instruction to keep abreast of developments. Richard Latham introduced himself as being an officer of the NYYC and a member of the NYYC America’s Cup Committee. At a meeting of the Committee on Sunday, August 21, 1983, he was asked to visit me at NSMB as soon as possible. The purpose of his visit was to find out “the truth relative to the role played by the National Aerospace Laboratory and NSMB in the design of *Australia II”*. He then produced two copies of a two-page document, one copy of which he handed to Dr. Rinus Oosterveld (my superior), also present at the meeting, and me. The title of the document had been crossed out but I could make out the following words: “Affidavit … by Dr. van Oossanen … on August 24, 1983”. The document can be described as containing a reasonably accurate account of what transpired at NSMB and NLR during April through August 1981. I no longer possess a copy of the document, but I possess a detailed memo, written that evening, of what it contained and what transpired at the meeting.

After the sentences in which I was described as knowledgeable and experienced in the art of Twelve Meter design as borne out by my papers on the subject in 1978 and 1981, the document told of the visits by Van Rijn and Valentijn to NLR and NSMB on August 9 and 10, respectively, from which they had learned about certain aspects of the design process which, together with other accounts, had resulted in the belief that *Australia II’s* hull and keel had not been designed by Ben Lexcen. The document then went on to briefly describe the following:

- Joop Slooff’s computer calculations
- That Joop Slooff first suggested the use of winglets
- That Ben Lexcen was the leader of the design team, consisting of between 6 and 8 people
- That NSMB produced the drawings from which the yacht was built
- That NSMB developed the software that was being used in Newport to monitor the yacht’s performance
- That NSMB was involved in the analyses of the yacht’s performance in Newport

I stated that the contents of the document were basically correct except for some minor points, but that I couldn’t sign the document for obvious reasons. Richard asked me to point out what minor points were incorrect, but I refused—mainly because Rinus Oosterveld asked me not to. I then raised the issue of why the NYYC would now be so determined to have *Australia II* disqualified when for the 1977 America’s Cup, Johan Valentijn basically designed the hull and keel of the original *Australia***.** I said that Johan wouldn’t have been an Australian national at the time. Richard refused to be drawn into a discussion of this nature.

I found Richard Latham’s position on the issue as it stood during the meeting as very reasonable. Although he asked me several times to pinpoint inaccuracies in the document, he remained polite and understanding. I felt a connection with him, and I think he did too. We would wave to each other afterward whenever *Black Swan* (Australia II’s tender with me on board to monitor the yacht’s sailing performance by means of a telemetry link) was in the neighborhood of the NYYC Committee vessel *Black Knight*.

### The Race

**Fig. 5:**
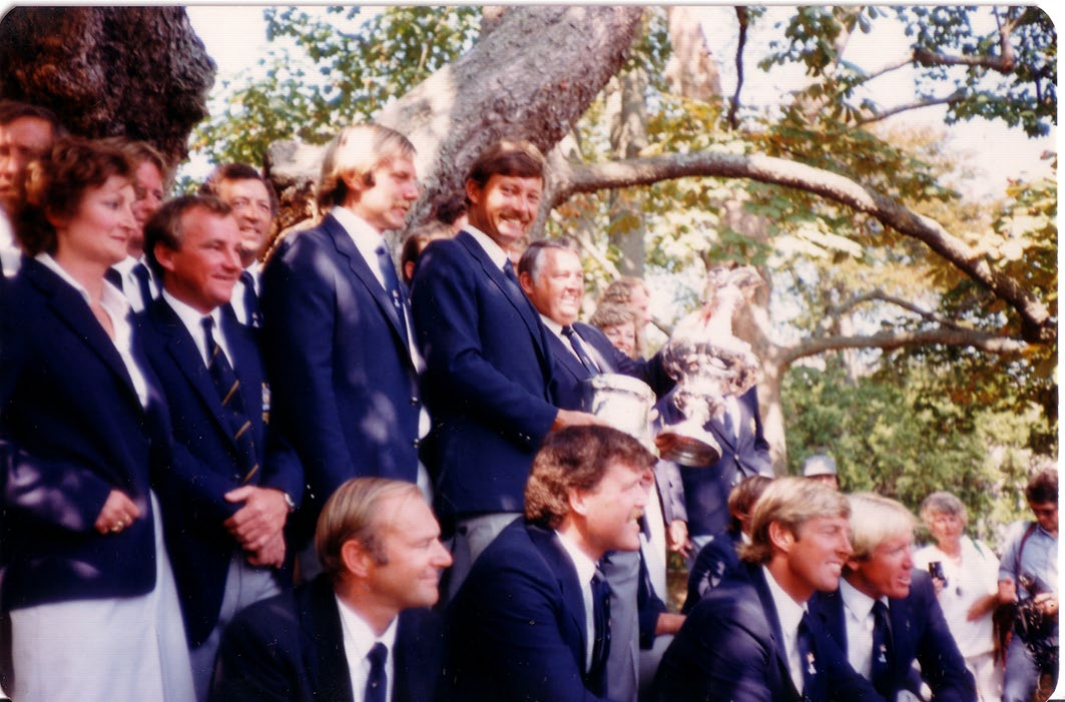
Photograph of the Australia II team at the America’s Cup presentation ceremony on September 27, 1983. PvO is in the front row closest to the camera

More than 400 journalists at Newport reported on every detail of the events as they occurred during that summer and when racing for the America’s Cup itself commenced most of the Western world listened and watched for the event to unfold in a best-of-seven-series. *Australia II* lost the first two races due to gear failure, then won the third, lost the fourth, and won the next three races to win by 4–3. The races were televised live to Australia and other countries (Fig. 5).

### Final Remarks

The discussion about inventorship in connection with the US patent early in 1983 resulted in me having to promise both Warren and Ben on March 18, 1983, to never mention our role and to always maintain that Ben was responsible for everything related to the design. I am on record as having said that many times when interviewed by journalists in Newport. However, we agreed during that meeting that if *Australia II* were to win the America’s Cup, he and Ben were to be mindful of our role when explaining to the media how they had won—thereby implying that I wanted them to exercise discretion when speaking with the media.

**Fig. 6:**
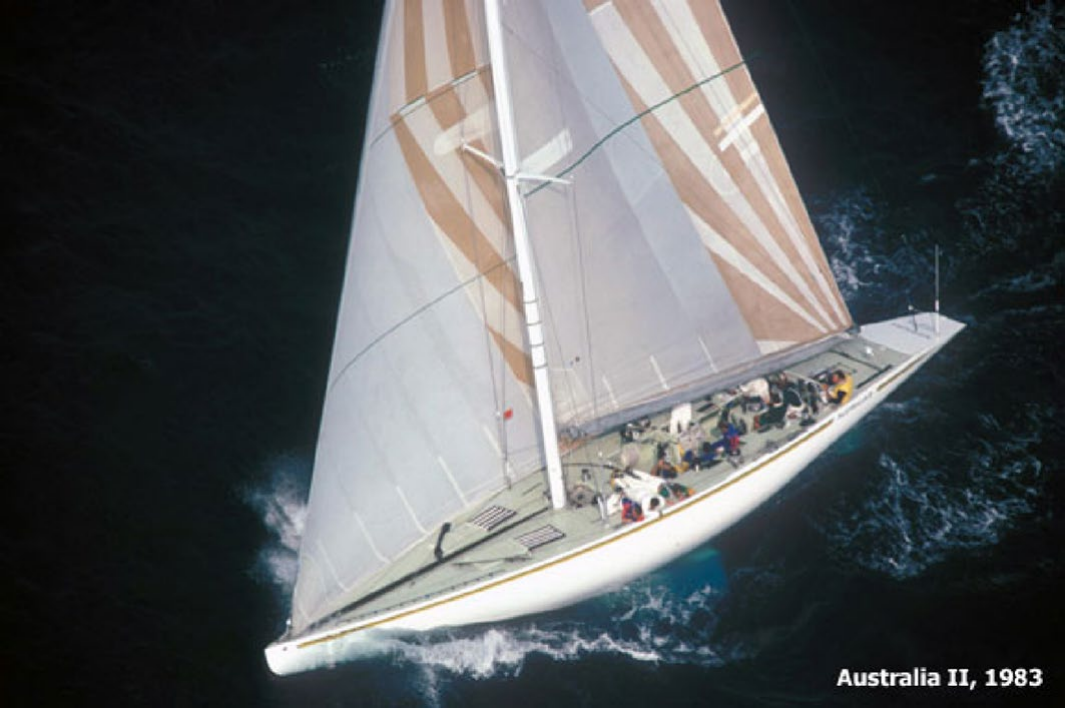
Australia II sailing in Newport

The question that haunts me to this day is what should I have done differently? I was aware of the nationality requirement the NYYC had imposed. Should I not have approached Alan Bond about being able to help him win the America’s Cup? The board of directors of NSMB told me it wasn’t our problem. Should I have read the willingness of NLR to assist differently? Joop Slooff had become a friend when I approached him about the project and I had no idea he would insist on recognition of his part in the project after having promised to keep what he was to do secret. His role was the cause of the nationality issue to flare up because of what he told Johan Valentijn on May 22, 1983. Should I not have promised Warren and Ben to keep the Dutch involvement a secret and to always maintain that Ben was the sole designer? When I promised that, I had no inkling of having to face dozens of journalists and film crews when in Newport who all wanted to know if I was involved in the design. It caused me to lie dozens of times. Should I have referred that question to Warren so as not to have to lie about it? Should I have disagreed more emphatically when Michael Boud wanted to have the patent drawn up? The patent issue caused Slooff to threaten to formally protest Ben Lexcen’s sole inventorship if he wasn’t to be included as inventor (Fig. 6).

Except for some presentations on the 1983 America’s Cup at local yacht clubs and articles in local media, I kept NSMB’s involvement secret until repeated articles about how Ben Lexcen designed *Australia II* started appearing around the time of Ben Lexcen’s induction into the America’s Cup Hall of Fame in 2006—presumably to support his nomination. Those articles originated in Australia and wouldn’t have been written had Warren Jones still been alive. I then decided to no longer remain quiet and when Dan Spurr, editor of *Professional Boatbuilder* visited our office in Wageningen in 2009, I decided to tell him of my involvement when he asked me about what had happened in 1983. That article made its way to the *Sydney Morning Herald*, one of Australia’s main newspapers, leading to a front-page article in the October 14, 2009 edition, entitled “The lie that captured the America’s Cup”. Scenes erupted such as those I experienced in Newport when journalists, TV and radio stations, wanted to ask me questions of what had happened in 1981. My staff and I answered every one of those questions diligently, providing supporting evidence where necessary. The difficulty in convincing some of Australia II’s crew is that they were present when I repeatedly stated that Ben Lexcen was the sole designer.

## Martin Peterson’s Ethical Analysis

The four most important ethical issues raised by the Australia II case seem to be the following: (i) Who should get credit for the innovative design of the wing keel and underwater body? (ii) Was it ethically permissible to exclude van Oossanen and Slooff from the patent application? (iii) Was it ethically permissible to conceal the truth about how the keel was designed for the public and other competitors? (iv) Do engineers have a professional obligation to respect the rules of sporting competitions such as the America’s Cup?

To answer these questions, we need a method. A straightforward option, which we will rely on here, is to apply a professional code of ethics to the facts of the case. However, it should be noted that mechanically applying a code of ethics to a real-world case is rarely sufficient for reaching a nuanced verdict. This is partly because different codes tend to yield conflicting recommendations. An alternative approach, preferred by some ethicists, is to instead apply one’s “favorite ethical theory” (such as consequentialism, Kantian duty ethics, or virtue ethics). However, different theories *also* tend to yield different recommendations—and there is no consensus on which theory is best. In this paper we note that professional codes typically do a good job of capturing the most pressing professional ethical issues for engineers, and we address the concern that different codes sometimes yield conflicting recommendations by applying an additional method presented in detail in Peterson (2017), in which moral paradigm cases are used as reference points for constructing a “moral map”. The application of this similarity-based method is admittedly somewhat sketchy in the present paper, but enough will be said to indicate how it could be applied to conflicts between different codes.

### Who Should Get Credit for the New Innovative Design?

The 1983 edition of the America’s Cup was sailed in the U.S., but the engineers involved in the project, van Oossanen and Slooff, were based in the Netherlands. This indicates that the American NSPE code and the Dutch KIVI code of ethics are the two most relevant documents to consider. NSPE stands for the *National Society for Professional Engineers*. It has about 35,000 members and its mission is to address the professional concerns of licensed engineers (called Professional Engineers, or P.E.s for short) across all engineering disciplines. KIVI stands for *Koninklijk Instituut Van Ingenieurs* (Royal Netherlands Society of Engineers) and it has about 17,000 members in the Netherlands.

According to the NSPE Code, “Engineers shall give credit for engineering work to those to whom credit is due, and will recognize the proprietary interests of others. … Engineers shall, whenever possible, name the person or persons who may be individually responsible for designs, inventions, writings, or other accomplishments.” (NSPE III.9.a) The KIVI code is somewhat less precise: “The engineer will not bring out misleading representations of the own work or of that of others, and he will sustain a reasonable level of effort in order to combat or correct misrepresentation by others.” (KIVI 5.4).

Ben Lexcen was listed as the sole inventor in the patent application submitted by his company Norport in February 1982. However, the evidence clearly shows that he was not the actual inventor.^7^ Lexcen had next to no input on the radical keel and underwater hull that was key for winning the Cup. The actual designers were van Oossanen and Slooff, so based on what we know today it is hard to deny that they deserve credit for their work. That said, it is worth stressing that van Oossanen’s and Slooff’s involvement increased gradually. They did not initially *intend* to redesign the entire keel and hull of the yacht, but after a couple of months of iterative testing and design that was what actually happened.

Should van Oossanen and Slooff receive full credit for the design? A possible compromise could be to credit the *entire team*, including Lexcen. However, if we believe that legal principles go hand in hand with moral ones, this is a nonstarter. As Doug Henderson (the patent lawyer) explained in the letter cited by van Oossanen, it is the individuals who *actually come up* with an innovative design who deserve (legal) credit: “if the leader of the team merely instructs the team members to do studies to find ways to improve a device, and a team member comes up with a specific feature which makes the device better, then the team member is the sole inventor.” This suggests that Lexcen should not get any (legal) credit for the design. The *ideas* and *technical solutions* were developed by van Oossanen and Slooff. It is hard to see why the same reasoning would not be applicable for settling the ethical issue.

The patent application never resulted in any commercially valuable patent. However, this seems irrelevant. Consider the following analogy: A researcher who plagiarizes a text is guilty of plagiarism even if the paper has no impact on the scientific community. It would be odd to maintain that the same would not apply to patent applications. By permitting his lawyers to submit a patent application in which he is falsely listed as sole inventor, Lexcen violated the same norm that a researcher violates when he or she submits a plagiarized paper for publication. The commercial value of the patent is irrelevant.

A complicating factor in the Australia II case might have been that van Oossanen at the time *agreed* to not being listed as an inventor in the patent application. Real-world cases are seldom black or white. However, even if van Oossanen and Slooff consented to not being mentioned, the deceptive nature of the patent application is hard to ignore. Arguably, as far as NSPE III.9.a is concerned (“Engineers shall give credit for engineering work to those to whom credit is due…”), it does not matter if those involved in the project consent to not getting credit. Those who *actually come* up with an innovative design should get credit for it, even if that was not what they wanted at the time. This is particularly important for inventions that are controversial or may harm people, such as weapons of mass destruction. In those cases (as opposed to sailboats) it is understandable that an inventor may not wish to get credit for a design, but it is nevertheless important for the history books to be truthful about the origins of major innovations.^8^

Another question that could be asked is whether van Oossanen and Slooff were obligated to *intervene* by, for instance, notifying the patent authorities about who the true inventors were. Interestingly, NSPE and KVI offer conflicting advice on this. The NSPE code merely requires engineers to intervene when *other engineers* violate the code (NSPE II.1.f). If a lawyer files an untruthful patent application, the lawyer does not violate the code simply because the code is not applicable to lawyers. Therefore, van Oossanen and Slooff had no obligation to report this. However, the KIVI code is more demanding. It states that engineers must “sustain a reasonable level of effort in order to combat or correct misrepresentation by others” (KIVI 5.4). This presumably includes misrepresentations by lawyers, meaning that it was not permissible to knowingly let other team members submit a patent application with false information. The term “reasonable level of effort” is of course vague, but it seems reasonable to think that this formulation would have required van Oossanen and Slooff to, for instance, make a stronger case for not submitting any patent application at all. However, it should also be noted that van Oossanen and Slooff are now, 40 years later, in compliance with the KIVI code as they both seem to be doing their duty to “combat or correct misrepresentation by others” by speaking openly about what actually happened.

### Was it Permissible to Keep the Truth About the Design Process Secret?

According to the NSPE code, engineers should “issue public statements only in an objective and truthful manner” and “avoid deceptive acts”. (NSPE I.3, I.5). The KIVI code states that engineers are not permitted to lie in “technical reports, depositions, and testimonies” (KIVI 3.1). This clause does not cover lies in public statements, but note that the KIVI code also states that “The engineer will not bring out misleading representations of the own [sic!] work or of that of others”. (KIVI 5.4) Lies are per definition misleading representations, so the only kind of lie not explicitly covered by the KIVI code are lies that do not concern *the work* of engineers.

Several members of the Australia II syndicate, including van Oossanen and Slooff, performed deceptive acts and issued false public statements. Although no team member lied or deceived anyone in “technical reports, depositions, and testimonies”, this violated NSPE I.3 and KIVI 5.4. However, it is worth to keep in mind that the consequences of telling the truth would have been dramatic. The Australian yacht would have been disqualified after millions of dollars had been spent on the campaign, and four decades later van Oossanen still believes that, “My family and I would not have been safe from vengeful Australians if I had [told the truth].”^9^

As explained by van Oossanen, Richard Latham flew to the Netherlands in the summer of 1983 on behalf of New York Yacht Club, hoping that van Oossanen would sign an affidavit explaining how the boat had been designed, which he refused to do. It is worth pointing out that van Oossanen would not have been permitted to sign the affidavit even if he had so wished because both the NSPE and KIVI codes emphasize that engineers must be loyal to their clients.^10^ Engineers are not permitted to cooperate with lawyers representing rival organizations without consent from their employers. Here is how this point is spelled out by the NSPE: “Engineers shall not disclose, without consent, confidential information concerning the business affairs or technical processes of any present or former client or employer, or public body on which they serve.” (NSPE III.4) This supports the conclusion that van Oossanen was not permitted to sign the affidavit without consent from the Australian syndicate *and* his supervisor Oosterveld. The KIVI code includes a similar provision: “The engineer will not disclose project information that is confidential without the consent of the parties, unless maintaining the confidentiality is contrary to the law or the good order.” (KIVI 2.6.1.). This formulation is somewhat more flexible as it makes it clear that “maintaining … good order” could in some cases be a reason for revealing confidential information. That said, it is far from evident that the provision was applicable to the Australia II case.

### Are Engineers Required to Respect the Rules of a Sporting Competition?

Under normal circumstances, it would *not* be unethical for an Australian sailor to ask a Dutch designer to design a boat, but the rules of the 1983 edition of the Cup stipulated that the designer had to come from the same country as the boat. There is no doubt that this rule was violated, but who should be held responsible for this?

No professional code I am familiar with includes special provisions that require engineers to comply with the rules of sporting competitions. However, engineers play a crucial role in many sports, including sailing, Formula 1 racing, skiing, and cycling. If an engineer performs an action that is unethical outside of a sporting competition, then that action is typically unethical also within the context of the competition. (A possible exception could be boxing: it is unethical to attack people on the street, but not in a boxing competition.) A more interesting question is whether actions that are permissible under *normal circumstances* are sometimes impermissible *because* the engineer is working for a sports team. As noted, KIVI 5.5 includes a somewhat vague prohibition against “dishonest practices”, but in the *Australia II* case it was not the engineering work that was dishonest, but rather the application (by non-engineers) of the new technology. The NSPE code is equally silent. The most relevant principle is the vague command that engineers shall, “conduct themselves honorably, responsibly, ethically, and lawfully” (NSPE 1.6), but in many real-world cases reasonable people can disagree on what that means.

In many sports, it is the athlete who is responsible for making sure the equipment used is compliant with the rules. A tennis player who competes with a racquet that is too big or too light will be disqualified even if the player did not know that the equipment was not compliant. In the *Australia II* case, it is not clear that all members of the Australian team knew that the most important parts of the boat had been designed by van Oossanen and Slooff. The Australian skipper John Bertrand knew the truth, but van Oossanen repeatedly assured the other crew members that Lexcen had designed the innovative underwater body. The analogy with the tennis player indicates that all this is irrelevant. The athletes are responsible for making sure that their equipment is compliant with the rules, even if they were unaware of what happened, or had the technical expertise required for doing this. Selecting good advisors is an essential skill for all athletes. Athletes have the *role responsibility* for using compliant equipment, even if they are not *causally responsible* for it.

### Ethics Beyond Professional Codes

A professional code of ethics can be useful for making an initial assessment of a complex case, but as noted earlier, mechanically applying a professional code is seldom sufficient for reaching a nuanced verdict. In the previous sections it has been demonstrated that although the NSPE and KIVI codes were both applicable to the Australia II case, they entail conflicting conclusions. For instance, the codes disagree on whether van Oossanen and Slooff were obliged to interven*e* as they found out that Lexcen would be listed as the sole inventor in the patent application. According to the NSPE code, they were not obliged to do so, but according to the KIVI code they were. Another example concerns whether it was permissible to lie to the public about the wing keel. The KIVI code prohibits engineers from lying or bringing out “misleading representations” under a wide range of circumstances, but not all. However, the NSPE code prohibits public lies of all forms. Because both codes have been written with great care, it is reasonable to think that this minor discrepancy reflects a deeper, underlying disagreement between NSPE and KIVI.

How should conflicts of this type be resolved? According to what one may call a minimalistic approach, ethicists should *suspend judgment* about issues on which professional codes yield conflicting advice. In those cases, we may instead revert to a purely legal assessment of the situation. A problem with the minimalistic approach is that it assumes that there is nothing more to professional ethics than what has been written down by a professional organization. However, professional codes are not the only possible justification for an ethical judgment. The minimalistic approach thus fails to account for the intuition that not all conflicts between codes are genuine ethical dilemmas that cannot be resolved. Another way to put the worry is to point out that the minimalistic approach presupposes a form of ethical nihilism—conflict cases have no solution—which is an implausible metaethical claim that few would be willing to accept without further qualifications, especially because new conflict cases could easily be created by adopting a new code.

Another method for resolving conflicts between codes is to appeal to some *additional* code (or principle). However, a problem with this is that the outcome would then depend heavily on the code selected for breaking the tie. This strategy merely moves the problem from one location to another. Instead of focusing on the conflict between the original codes, we would have to focus on what additional code we should apply. It is hard to see how we can resolve conflicts between codes in a nonarbitrary way by simply drafting a new document. Doing so seems to undermine the original documents, or at least make them redundant.

A better proposal is, it seems, to analyze conflicts by considering how *similar* they are to cases we already know how to analyze. The *locus classicus* for the similarity-based approach to applied ethics is Aristotle's principle of formal justice, which asserts that we should "treat like cases alike" (NE 1131a10-b15). Christian ethicists have developed Aristotle’s insight into a controversial method of applied ethics called casuistry.^11^ Casuists extend Aristotle’s principle to cases that are not fully similar in all morally relevant aspects. In their view, we should assess a choice situation by comparing it to a set of paradigm cases, i.e. cases we are familiar with and know how to analyze. A stock objection to this is that the casuist method is too imprecise to be practically useful. Another objection is that it fails to acknowledge the importance of moral principles in applied ethics. Peterson (2017) seeks to address these shortcomings by combining Aristotle’s notion of morally relevant similarities with the theory of conceptual spaces.^12^ In this approach, paradigm cases are used as reference points in a “moral map”: If a case we wish to analyze (for instance, a case in which two professional codes yield conflicting verdicts) is more similar to one paradigm case rather than another, then the same moral principle that applies to the first paradigm case is also applicable to the new case. In this manner, moral principles are given a key role in similarity-based reasoning in applied ethics. Moreover, by applying the formal machinery of the theory of conceptual spaces, this “geometric” approach to applied ethics can be formalized and worked out in great detail, meaning that this method is no less precise than other methods of applied ethics: If two cases, *x* and *y*, are fully similar in all morally relevant respects, and if some principle *p* is applicable to case *x*, then *p* is also applicable to *y*. Furthermore, if case *x* is more similar to paradigm case *w* than to paradigm case *v*, and *p* is applicable to *w*, then *p* is also applicable to *x*.^13^

When applying the similarity-based approach to the issue of whether it was permissible to lie to the public about the wing keel (an issue the KIVI and NSPE codes analyze differently), it is helpful to note that people who participate in sporting competitions are typically permitted to lie about *some* matters that pertain to the competition. For instance, soccer players are permitted to lie to the press about their expectations for an upcoming game. If they had been required to always tell the truth to the press, that could give the other team an advantage, which would be a more important consideration in this type of case. This could serve as a paradigm case for a choice situation in which innocent lies can be ethically permissible in sporting competitions. However, lies that fundamentally impact the integrity of the game itself are of course paradigmatically impermissible, such as lies about the material of some piece of equipment. By comparing how similar the lies uttered by the Australia II team members were to these two types of paradigm cases, we may conclude that the lies uttered by the Australia II team members were more similar to the innocent kind of lies (they lied about who had done what, not about materials or other properties of the boat itself) and therefore morally permissible. This suggests that we should side with the KIVI code rather than the NSPE code.

Let us also consider whether van Oossanen and Slooff had any obligation to intervene when it became clear that the patent application falsely listed Lexcen as the sole inventor of the wing keel. It is helpful to note that there is a wide class of minor rule transgressions that would not trigger any obligation for bystanders to intervene. You are, for instance, not obliged to report the fact that a player in a soccer game touched the ball with his hands, it is up to the referee to notice that. Cases of this type can serve as paradigm cases for a principle of non-intervention. However, there is also a wide class of very different cases in which bystanders *are* required to intervene. For instance, in a typical case of scientific misconduct anyone with knowledge of a plagiarized publication would typically be obliged to notify the journal’s editor. The patent case is arguably more similar to a plagiarism case than to a minor, undetected rule violation in a soccer game. If so, it follows that van Oossanen and Slooff had an obligation to intervene by notifying the patent office, albeit not a very strong one as the stakes were lower than in some cases involving errors in scientist publications, in which people’s lives may ultimately be at stake. The upshot is that we should once again side with the KIVI code rather than the NSPE code.

## Discussion Questions

This article is likley to be used for educational purposes, so it is worthwhile to formulate a couple of discussion questions raised by the Australia II case. We advise the reader to discuss the following questions with a fellow student or engineer:

1. Imagine that you are van Oossanen or Slooff. Would you have acted differently? If so, why and how? What would you have done when you realized that the rules of the America’s Cup had been violated? Do you think it would have been better to reveal the truth despite the negative consequences of doing so? Keep in mind that millions of dollars had already been invested. Does it matter if the competing teams also violated the rules?
2. Neither van Oossanen nor Slooff intented to violate the rules of the America’s Cup at the outset. The transgression happened gradually as the keel and hull were modified in an iterative design process. Was there a *precise point* at which the transgression occurred, or was there a gray area in which it was neither true nor false that a transgression had occurred? Can ethically relevant principles be vague?
3. Are the principles of a professional code of ethics binding under all circumstances? Is it always wrong to lie even if that has the best consequences for everyone?
4. Can ethics be codified? If “yes”, what gives a code its normative force? In *virtue of what* are the principles stated in the NSPE and KIVI Codes valid? If “no”, how do we avoid the conclusion that “anything goes”?
5. What should engineers do if two or more equally applicable professional codes give conflicting advice about what one ought to do?

## Conclusion

In the Australia II case, the most significant ethical violation occurred when a patent application for the revolutionary wing keel was submitted by the Australia team knowing full well that Lexcen was not the actual inventor. Some of the other violations, such as the manner in which the public was deceived, were somewhat less important. The U.S. patent application was eventually withdrawn, but it is important for the history books that we give credit to those to whom credit is due. By presenting several new details about how the wing-keel was designed, we have offered additional nuances to this fascinating case.

## Footnotes

1 The interview in which Mr. Hawke makes this statement is available on YouTube: https://youtu.be/LB0WvpiUA_I (Accessed Feb. 5, 2023).

2 Way and Way (2022), Britannica (2023).

3 Lexcen and Norport (1982), Pat. AU8566882A. The patent process was never completed.

4 See "Ben Lexcen" in *Encyclopedia Britannica*. That Lexcen did not know how to operate a computer was witnessed by van Oossanen in the summer of 1981.

5 See e.g. Fishman (1983a), Lloyd (1985), Fenely (2009), and Spurr (2009).

6 Van Oossanen was interviewed about the wing keel by Spurr (2009).

7 See, for instance, Spurr (2009) and Way and Way (2022).

8 A reviewer has pointed out that it might be reasonable to sometimes let the inventor’s preference for anonymity trump NSPE III.9.a. However, as our focus is on applying the code as it is currently written, we will refrain from proposing any claim about how it could be modified to accommodate such exceptions.

9 Email from van Oossanen to Peterson, March 4, 2024.

10 Note that this principle is not absolute. If, for instance, the obligation to be loyal clashes with an imminent threat to public health and safety, the obligation to be loyal could potentially be set aside.

11 For an over view, see Jonsen and Toulmin (1988).

12 See Gärdenfors (2004, 2014).

13 For a detailed presentation and discussion, see Peterson (2017).
